# Effects of Shenling Baizhu powder on pyrotinib-induced diarrhea: analysis of gut microbiota, metabonomics, and network pharmacology

**DOI:** 10.1186/s13020-022-00696-3

**Published:** 2022-12-17

**Authors:** Jingjiang Lai, Fengxian Jiang, Xiaoli Zhuo, Xiaoying Xu, Lei Liu, Ke Yin, Jingliang Wang, Jing Zhao, Wei Xu, Hongjing Liu, Xuan Wang, Wen Jiang, Ke Wang, Shuping Yang, Honglin Guo, Fanghua Qi, Xiaotian Yuan, Xiaoyan Lin, Guobin Fu

**Affiliations:** 1 Department of Oncology, Shandong Provincial Hospital Affiliated to Shandong First Medical University, Jinan, 250021 People’s Republic of China; 2 The Second Clinical Medical College, Shandong University of Traditional Chinese Medicine, Jinan, 250002 People’s Republic of China; 3 The Clinical Medical College, Shandong First Medical University (Shandong Academy of Medicine), Jinan, 250117 People’s Republic of China; 4 Department of Oncology, Shandong Provincial Hospital Cheeloo College of Medicine, Shandong University, Jinan, 250021 People’s Republic of China; 5 Laboratory Animal Center, Shandong Provincial Hospital Affiliated to Shandong First Medical University, Jinan, 250021 People’s Republic of China; 6 Department of Pathology, Shandong Provincial Hospital, Cheeloo College of Medicine, Shandong University, Jinan, 250021 People’s Republic of China; 7 Department of Pathology, Shandong Provincial Hospital Affiliated to Shandong First Medical University, Jinan, 250021 People’s Republic of China; 8 Department of Central Laboratory, Shandong Provincial Hospital, Cheeloo College of Medicine, Shandong University, Jinan, 250021 People’s Republic of China; 9 Department of Biostatistics, School of Public Health, Cheeloo College of Medicine, Shandong University, Jinan, 250021 People’s Republic of China; 10 Traditional Chinese Medicine, Shandong Provincial Hospital affiliated to Shandong First Medical University, Jinan, China

**Keywords:** Shenling Baizhu powder, Pyrotinib-induced diarrhea, Mechanism, Gut microbiota, Metabonomics, Network pharmacology

## Abstract

**Background:**

Shenling Baizhu Powder (SBP) is a traditional Chinese medicine (TCM) prescription, which has the good efficacy on gastrointestinal toxicity. In this study, we used gut microbiota analysis, metabonomics and network pharmacology to investigate the therapeutic effect of SBP on pyrotinib-induced diarrhea.

**Methods:**

24 Rats were randomly divided into 4 groups: control group, SBP group (3.6 g/kg /bid SBP for 10 days), pyrotinib model group (80 mg/kg/qd pyrotinib) and pyrotinib + SBP treatment group. A 16S rRNA sequencing was used to detect the microbiome of rat fecal bowel. Metabolic profiles were collected by non-targeted metabolomics and key metabolic pathways were identified using MetaboAnalyst 5.0. The antitumor effect of SBP on cells treated with pyrotinib was measured using a CCK-8 assay. Network pharmacology was used to predict the target and action pathway of SBP in treating pyrotinib-related diarrhea.

**Results:**

In vivo study indicated that SBP could significantly alleviate pyrotinib-induced diarrhea, reaching a therapeutic effect of 66.7%. SBP could regulate pyrotinib-induced microbiota disorder. LEfSe research revealed that the SBP could potentially decrease the relative abundance of *Escherichia, Helicobacter* and *Enterobacteriaceae* and increase the relative abundance of *Lachnospiraceae, Bacilli, Lactobacillales *etc. In addition, 25-Hydroxycholesterol, Guanidinosuccinic acid, 5-Hydroxyindolepyruvate and cAMP were selected as potential biomarkers of SBP for pyrotinib-induced diarrhea. Moreover, Spearman's analysis showed a correlation between gut microbiota and metabolite: the decreased 25-hydroxycholesterol in the pyrotinib + SBP treatment group was negatively correlated with *Lachnospiraceae* while positively correlated with *Escherichia* and *Helicobacter*. Meanwhile, SBP did not affect the inhibitory effect of pyrotinib on BT-474 cells and Calu-3 cells in vitro. Also, the network analysis further revealed that SBP treated pyrotinib-induced diarrhea through multiple pathways, including inflammatory bowel disease, IL-17 signaling pathway, pathogenic *Escherichia coli* infection and cAMP signaling pathway.

**Conclusions:**

SBP could effectively relieve pyrotinib-induced diarrhea, revealing that intestinal flora and its metabolites may be involved in this process.

**Supplementary Information:**

The online version contains supplementary material available at 10.1186/s13020-022-00696-3.

## Introduction

Pyrotinib is an irreversibly targeted small molecule tyrosine kinase inhibitor developed in China. It targets epidermal growth factor receptor 1 (EGFR) and human epidermal growth factor receptor 2 (HER2) and has shown to be effectively against HER2-positive breast cancer and lung cancer [1–5]. Yet, current breast cancer studies suggested that pyrotinib combined with capecitabine has a 95% incidence of diarrhea [2]. Pharmacoeconomic analyses showed that severe diarrhea requiring hospitalization adds about $25,000 to health care costs [6], which increases the financial burden of patients. In addition, diarrhea not only affects patients' quality of life but can also lead to dehydration and electrolyte disorders and may even be life-threatening. Thus, patients with diarrhea are often forced to lower the drug dose or stop taking drugs altogether, which negatively affects treatment effectiveness [7].

There is a strong correlation between the occurrence of diarrhea and intestinal microflora disorder [8, 9]. Studies have shown that the excessive proliferation of pathogenic bacteria and the accumulation of toxins can lead to mucosal inflammation and increased epithelial cell barrier permeability, which in turn may lead to diarrhea [10, 11]. Metabolites and small molecules produced by the gut microbiome are absorbed by the host intestine and may affect the host's metabolism [12]. Metabolic disorders have proven to be closely related to diabetes, cancer, inflammation, and many other diseases. Therefore, biomarkers related to disease diagnosis can be found by comparing metabolite differences between body physiology and disease states [13]. Recently, the gut microbiome has become a novel and important area for understanding Traditional Chinese medicine (TCM) [14]. Compelling evidence supports the hypothesis that interactions between TCM and gut microbiota may lead to changes in microbiota and metabolic components [15]. In addition, network pharmacology has been widely and effectively applied in studying the mode of action of TCM formulae [16, 17]. In this sense, systems biology strategies are highly valued for their potential to elucidate TCM syndrome mechanisms by integrating multi-omics approaches and network pharmacology.

Shenling Baizhu Powder (SBP) is a classic traditional Chinese formulation composed of ginseng, atractylodes, poria cocos, yam, lotus seed meat, white lentil bean, coix seed, amomum, platycodon grandiflorum, and stir-fried licorice, as listed in Table 1 [18, 19]. This means that SBP has a compelling safety profile because it has been used by humans for thousands of years. SBP can strengthen the spleen and yiqi and cease diarrhea. In clinical practice, SBP has been used to treat chronic diarrhea, gastrointestinal dysfunction, and other diseases [20, 21]. It can also reduce gastrointestinal toxicity and side effects caused by radiotherapy and chemotherapy [22]. Thus, discovering anti pyrotinib-induced diarrhea agents from TCM may provide an idea for developing anti pyrotinib-induced diarrhea drugs with both effectiveness and safety.

**Table 1:** Components of Shenling Baizhu powder

Chinese name	English name	Latin name of plants	Family	Medical part
Ren Shen	Ginseng	*Panax ginseng* C.A.Mey	*Araliaceae*	root and rhizome
Fu Ling	Poria	*Poria cocos (Schw.)* Wolf	*Polyporaceae*	sclerotium
Bai Zhu	Atractylodes Rhizome	*Atractylodes macrocephala* Koidz	*Compositae*	rhizome
Shan Yao	Dioscorea Rhizome	*Dioscorea opposita* Thunb	*Dioscoreaceae*	rhizome
Bai Bian Dou	Dolichos Seed	*Dolichos lablab* L	*Leguminosae*	seed
Lian Zi	Nelumbo Seed	*Nelumbo nucifera* Gaertn	*Nelumbonaceae*	seed
Yi Yi Ren	Coix Seed	*Coix lacryma-jobi L. var. ma-yuen.* (Rom.Caill.) Stapf	*Poaceae*	kernel
Sha Ren	Amomum Fruit	*Amomum villosum* Lour	*Zingiberaceae*	fructus
Jie Geng	Platycodon Root	*Platycodon grandifforus* (Jacq.) A. DC	*Campanulaceae*	root
Gan Cao	Glycyrrhiza	*Glycyrrhiza uralensis* Fisch	*Leguminosae*	root and rhizome

Therefore, the present study aimed at assessing the therapeutic effect of SBP on pyrotinib-induced diarrhea and studying its possible mechanisms of action. Briefly, in this study, we established a pyrotinib-induced diarrhea model to explore the effect of SBP on pyrotinib-induced diarrhea, and then combined intestinal flora, metabolomics and network pharmacology to further explore the mechanism of SBP in alleviating pyrotinib-induced diarrhea. Overall, this strategy compensates for the lack of experimental validation in network pharmacology and the lack of upstream molecular mechanisms and drug binding targets in metabolomics, which contributes to a better understanding of the therapeutic mechanism of SBP in alleviating pyrotinib-induced diarrhea. The findings will provide scientific and rigorous proof of the potential applications of SBP as a complementary therapy to pyrotinib-induced diarrhea.

## Materials and methods

### Reagents and materials

Human cancer cells (BT-474 and Calu-3) were bought from the Wuhan puno game life science and technology co., LTD. HER2 antibody was Proteintech Group, Inc. Pyrotinib API (powder) was provided by Jiangsu Hengrui Medicine Co., Ltd. SBP (20170316) was obtained from Shanxi Huakang Pharmaceutical Co., LTD. (National Drug License No. Z14020346) and its quality control was implemented according to the Standards of “Pharmacopoeia of the People’s Republic of China (2015 edition) [19]. SBP contains the 10 Chinese medicines and it was checked using the http://www.theplantlist.org database. Besides, HPLC characteristic fingerprint of the this batch SBP (number 20170316) was studied by *Liu and Zhu* et al. and the level of ginsenoside Rg1, ginsenoside Rb1, ginsenoside Re, glycyrrhizin and glycyrrhizic acid was 3.15, 1.81, 1.70, 1.11 and 4.67 mg/g respectively [23].

### Cell culture and cell growth curves

BT-474 cells were cultured in DMEM medium containing 10% fetal bovine serum and 1% double antibiotic (penicillin, streptomycin); Calu-3 cells were cultured in MEM medium containing 10% fetal bovine serum and 1% double antibiotic (penicillin, streptomycin). The two cell lines were cultured in a humidified atmosphere containing 5%CO_2_/95% air at 37 °C.

In this experiment, cells in the logarithmic growth phase and good condition were tested. Briefly, cells were inoculated into the 96-well culture plate at a density of 3000 cells/well. After cell adherence, cells were exposed to the complete medium containing SBP or pyrotinib or pyrotinib + SBP or DMSO (control group) for 1, 2, 3, 4, and 5 days to examine the cell growth. At each time point, 100 μl CCK-8 solution was added to each well and incubated for 2 h at 37 °C. The absorbance at 450 nm was determined using a microplate reader. All experiments were conducted in triplicate.

### Animals and the model of pyrotinib-induced diarrhea

Healthy Female Wistar rats (6 weeks old) were purchased from Sberford Company. All the animals were housed in the environment with a temperature of 22 ± 1 °C, relative humidity of 50 ± 1%, and a light/dark cycle of 12/12 h. All animal studies (including the ratseuthanasia procedure) were done in compliance with the regulations and guidelines of the animal ethics committee of the Shandong Provincial Hospital Affiliated to Shandong First Medical University, and conducted according to the AAALAC and the IACUC guidelines (NO.SDNSPC2021-0153).

The rats were raised in a single cage, and the experiment began after one week of adaptive feeding. Before the experiment, 24 rats were randomly divided into 4 groups (6 rats in each group): control group, SBP group, pyrotinib model group and Pyrotinib + SBP treatment group. The experiment lasted for 10 days. Based on our previous studies, pyrotinib was modeled at a dose of 80 mg/kg once daily, and the dose of SBP was 3.6 g/kg twice a day. The control group was given the same amount of saline.

The diarrhea rat model was established based on the previously described approach. Briefly, rats were given 80 mg/kg pyrotinib daily, and varying degrees of diarrhea persisted for 3–4 days.

### Enteric toxicity test

Intestinal tissue samples from different sites were collected and fixed in 4% formaldehyde solution, dehydrated with a series of ethanol solutions, paraffin-embedded, sectioned (4–5 μm), stained with hematoxylin and eosin (HE), and then observed under the Olympus BH22 microscope (Japan) as previously described. In addition, the degree of the damage was scored by two investigators using a perfect histological standard system.

### 16S rRNA gene sequence analysis

As previously mentioned, the samples were sent to Suzhou Taihe Biotechnology Co., Ltd (China) for DNA extraction and 16S ribosomal RNA (rRNA) gene region analysis. And the hypervariable region of the 16S rRNA gene V3-V4 was PCR amplified using the primers 357F (5′-ACTCCTACGGRAGGCAGCAG-3′) and 806R (5′-GGACTACHVGGGTWTCTAAT-3′). Subsequently, PCR amplification was performed to construct the Miseq library. Reads obtained from Miseq sequencing were first spliced according to overlap relationships. The sequence quality was controlled and filtered, and OTU cluster analysis and species taxonomic analysis were performed after distinguishing samples. Alpha diversity analysis was performed using Chao 1, Ace, Shannon, and Simpson indices while beta diversity analysis was performed using PCoA (Principal coordinates analysis). The analysis of these diversity indices is via QIIME2 (https://qiime2.org/). The Student’s t test, LEfSe and other statistical analysis methods were conducted to test the significance of species composition and community structure. Besides, LEfSe analysis results included three parts, which were histogram of LDA value distribution of significantly different species. Taxonomists branching diagram (Cladogram) and abundance histogram between groups, the results of the analysis through the Galaxy online platform (http://huttenhower.sph.harvard.edu/galaxy/) analysis.

### HPLC-MS/MS analysis

The intestinal tissues of rats were delivered to Suzhou Panomico Biomedical Technology Co., Ltd (China) for metabonomics analysis. The LC analysis was performed on a Vanquish UHPLC System (Thermo Fisher Scientific, USA). Chromatography was carried out with an ACQUITY UPLC^®^ HSS T3 (150 × 2.1 mm, 1.8 µm) (Waters, Milford, MA, USA). The column maintained at 40 ℃. The flow rate and injection volume were set at 0.25 mL/min and 2 μL, respectively. Mass spectrometric detection of metabolites was performed on Orbitrap Exploris 120 (ThermoFisher Scientific, USA) with ESI ion source. Briefly, 250 mg of tissue samples were placed in Eppendorf (EP) tubes and re-suspended in precooled 80% methanol and 0.1% formic acid. The samples were then incubated on ice for 5 min and centrifuged at 15000 g for 20 min at 4 °C. The samples were then transferred to a new EP tube and centrifuged at 15000 g for 20 min at 4 °C. The supernatant was then injected into liquid chromatography-tandem mass spectrometry (LC-MS/MS) system for analysis. After scaling data, models were built on principal component analysis (PCA), orthogonal partial least square discriminant analysis (OPLS-DA), and partial least-square discriminant analysis (PLS-DA). The reliability of PLS-DA or OPLS-DA model should be validated by the permutation test. Finally, metabolites with P value  < 0.05 and VIP values  > 1 were considered as statistically significant ones.

### Network pharmacology construction

#### SBP drug target and diarrhea target screening

Systematic pharmacology of Traditional Chinese Medicine (TCMSP) was used to set the active ingredient compounds of SBP required for screening with oral bioavailability  ≥ 30% and drug similarity  ≥ 0.18, which is the potential drug target of SBP. Drugbank and Genecard databases were used to screen pathological targets associated with pyrotinib-induced diarrhea. Arrive TCMSP can click https://tcmspw.com/tcmsp.php. The Venn diagram evaluated all major targets of SBP and pyrotinib-induced diarrhea to identify potential targets of SBP for improving pyrotinib-induced diarrhea.

#### Selection of the best targets for SBP to improve pyrotinib-induced diarrhea and the construction of the related network

The STRING database was used to obtain target-to-target function-related protein networks, protein-protein interaction (PPI) networks, and TSV data. TSV data were topologically analyzed using CytoNCA in Cytoscape3.7.2.

#### Analysis of functional processes and molecular pathways

The effective targets of SBP were uploaded to the David database for functional enrichment analysis of gene ontology (GO), including biological process and molecular function analysis. Meanwhile, Kyoto Encyclopedia of Genes and Genomes (KEGG) pathway enrichment analysis was performed to obtain the main pathway of SBP in pyrotinib-induced diarrhea. Cytoscape3.7.2 software was used for the visualization of network relationships.

### Co-occurrence network analysis

To understand the correlation between metabolites and different genera or species, we constructed a co-occurrence network based on 16S rRNA and HPLC-MS/MS data. According to the relative abundance of each species/genus, a co-occurrence network was constructed using Spearman correlation coefficient to analyze fecal gut microbiome and host intestinal tissue metabolites. The final result was visualized by a heat map.

### Data statistics and analysis

SPSS 22.0 software was used for statistical analyses. The measurement data were analyzed by unpaired, two-tailed t test. The enumeration data were analyzed by the Chi-squaretest or Fisher’s exact test. *P* < *0.05* was considered statistically significant.

## Results

### Therapeutic effect of SBP on pyrotinib-induced diarrhea

**Fig. 1:**
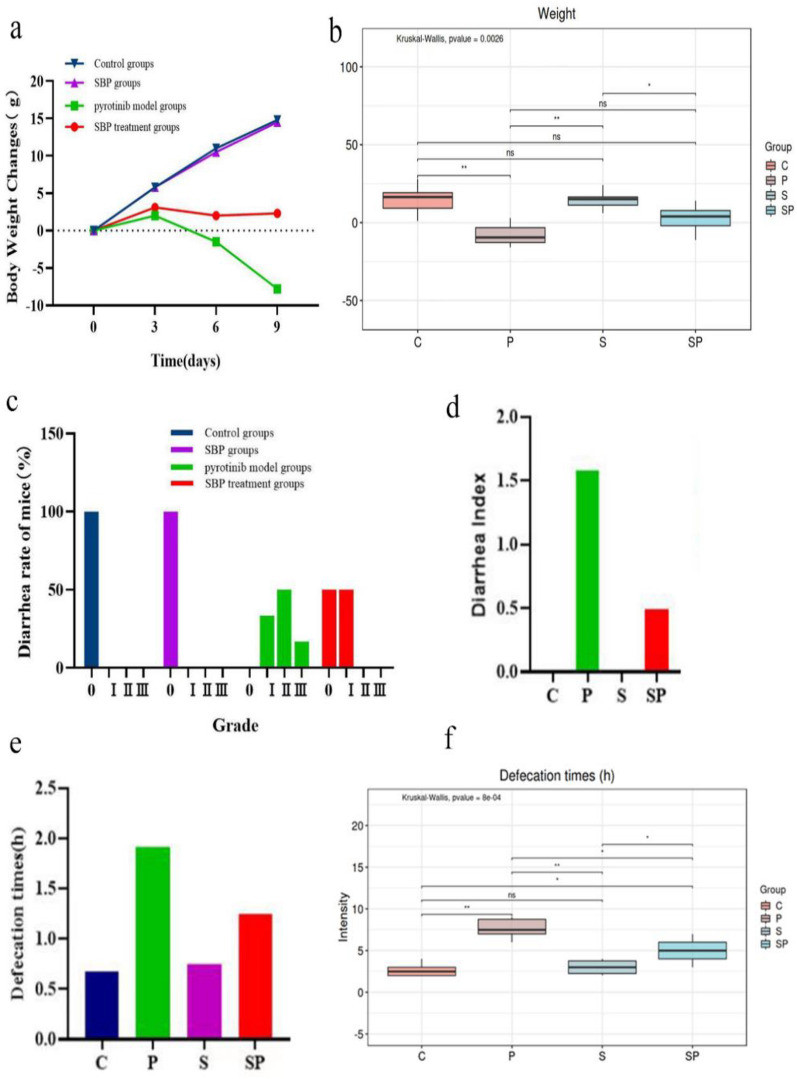
Diagram of weight change and diarrhea assessment in rats. **a**–**f** Body weight change, diarrhea grade, defecation frequency, and diarrhea index chart of rats in the control group, the SBP group, the pyrotinib model group, and the pyrotinib + SBP treatment group. *SBP* Shenling Baizhu Powder. *P < 0.05; **P < 0.01; ***P < 0.001

From day 1 to 3, the average body weight of rats in each group showed an increasing trend, where the weight gain of rats in the control group was the most obvious, while the weight gain of rats in the pyrotinib group was the lowest. From day 4 to 9, the weight of rats in the control group and the SBP group continued to increase, and the range was significant, while the weight of rats in the pyrotinib model group gradually decreased, and the range was significant (the difference between the pyrotinib model group and the control group was statistically significant, p < 0.001) (Fig. 1a, b).

On the last day of the study, the efficacy of SBP on the treatment of pyrotinib-induced diarrhea was evaluated according to the defecation times (/h), diarrhea grade, and diarrhea index in rats [24, 25]. The results showed that compared with the control group, the rats in the pyrotinib model group showed increasing defecation frequency and loose stool and had grade II diarrhea (the difference was statistically significant, *p* < *0.001*). Compared with the pyrotinib model group, the frequency of defecation in the pyrotinib + SBP treatment group decrease, and the rats had mild diarrhea, namely grade I diarrhea (the difference was statistically significant, *p* < *0.001*) (Fig. 1c, e–f). Meanwhile, we found that the diarrhea index of the pyrotinib model group was significantly higher than that of the control group*;* the diarrhea index of the SBP group was higher than that of the control group but significantly lower than that of the pyrotinib group (Fig. 1d). Based on these studies, we concluded that SBP was effective in alleviating pyrotinib-induced weight loss and diarrhea.

### Antitumor effect of SBP on the cells treated with pyrotinib in vitro

**Fig. 2:**
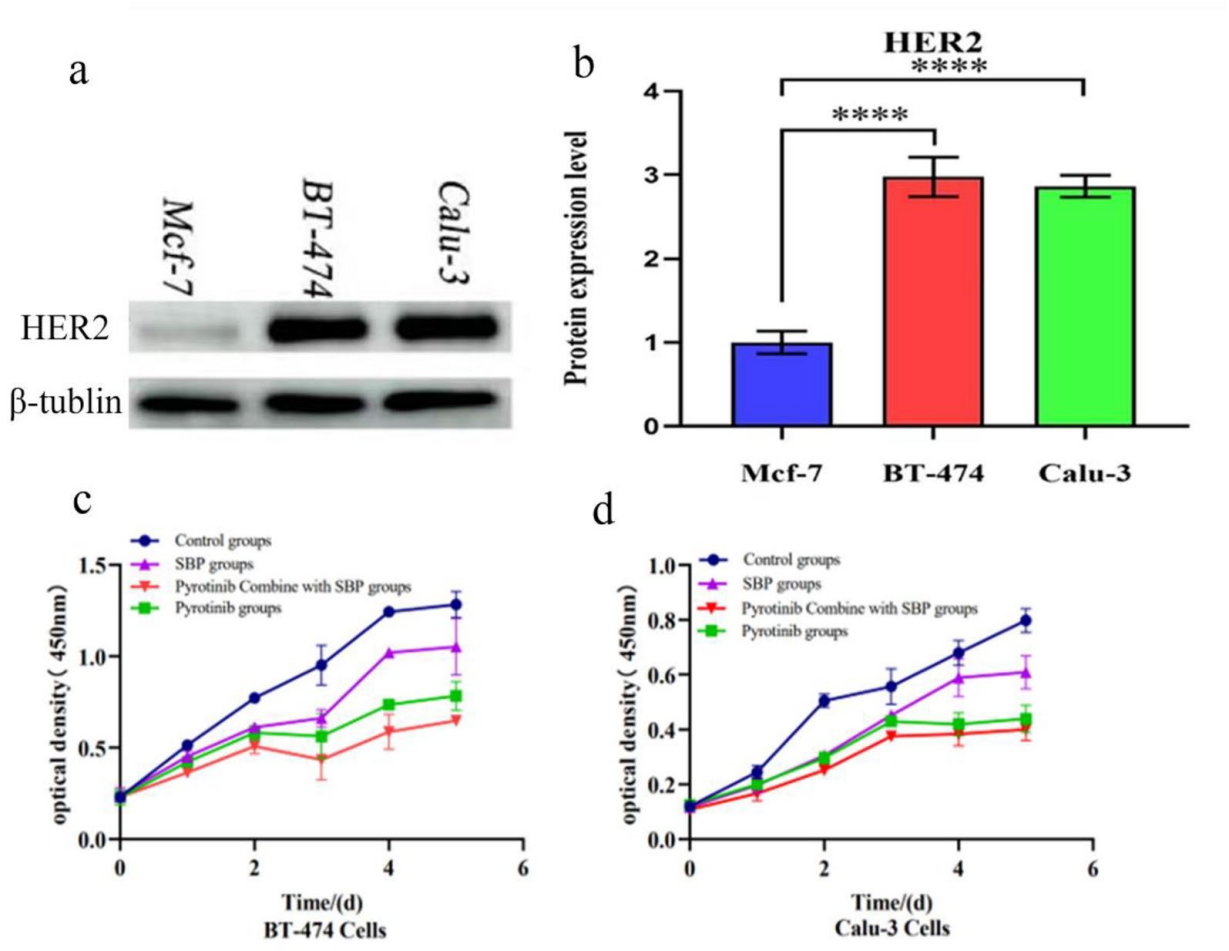
HER2 expression and the growth curve of BT-474and Calu-3 cells. **a**–**b** High HER2 expression in BT-474 and Calu-3 cells. **c**–**d** The growth curves of HER2-overexpressing human breast cancer cell line BT-474 and human lung cancer cell line Calu-3, respectively. *P < 0.05; **P < 0.01; ***P < 0.001

To further clarify the effects of SBP on the antitumor efficacy of pyrotinib, HER2-overexpressed human breast cancer cell line BT-474 and human lung cancer cell line Calu-3 were selected for cell experiments [26, 27]. Compared with MCF-7 cells (A cell with low HER2 expression level was detected as positive WB by using HER2 Polyclonal antibody (18299-1-AP)), HER2 expression level was significantly higher in BT-474 and Calu-3, confirming that HER2 was overexpressed in the two cell lines used in our experiment (Fig. 2a, b). Then, in our study, considering that the tested drug Shenling Baizhu Powder has mild drug properties, we selected 0,1,2,3,4,5 days as time points to observe the effects of the drug on cell viability when cell growth allowed. We found that the cells in the control group grew well (OD value showed an increasing trend over time). Compared with the control group, cell proliferation was all reduced in SBP group, pyrotinib group and pyrotinib + SBP group, but the magnitude of their reduction was different, indicating that they had different degrees of inhibition on cell growth. Yet, compared with the pyrotinib group, the proliferation rate of the cells in pyrotinib + SBP group decreased, indicating that indicating that SBP combined with pyrotinib had better antitumor efficacy than pyrotinib alone. (Fig. 2c, d).

### Protective effect of SBP on pyrotinib-induced pathological injury in rats

**Fig. 3:**
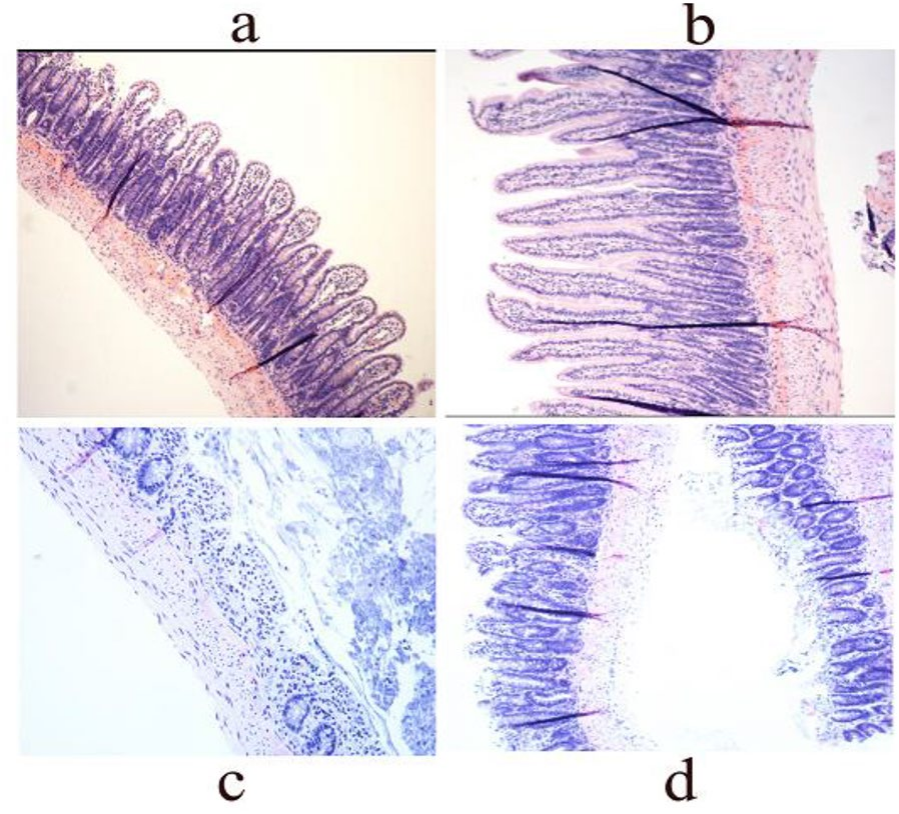
Pathological section of intestinal mucosa of rats. **a**–**d** Intestinal mucosal tissues of rats in the control group **a**, the SBP group **b**, the pyrotinib model group **c**, and the pyrotinib + SBP treatment group **d**, Hematoxylin-eosin staining, Image magnification 100 ×

We evaluated the histological changes of the ileum in each group, andthe pathological changes of the ileum are shown in Fig. 3. The ileum mucosa displayed normal histological features in the control group, while the ileum mucosa erosion was accompanied by persistent inflammatory cell infiltration, mucosal hyperemia, and inflammatory alterations in the pyrotinib model group. Besides, the features of the mucosa of the pyrotinib + SBP treatment group were identical to those of the control group. The results demonstrated that SBP prevented mucosal damage caused by pyrotinib.

### Gut microbiome analysis

#### SBP regulates microbial diversity

**Fig. 4:**
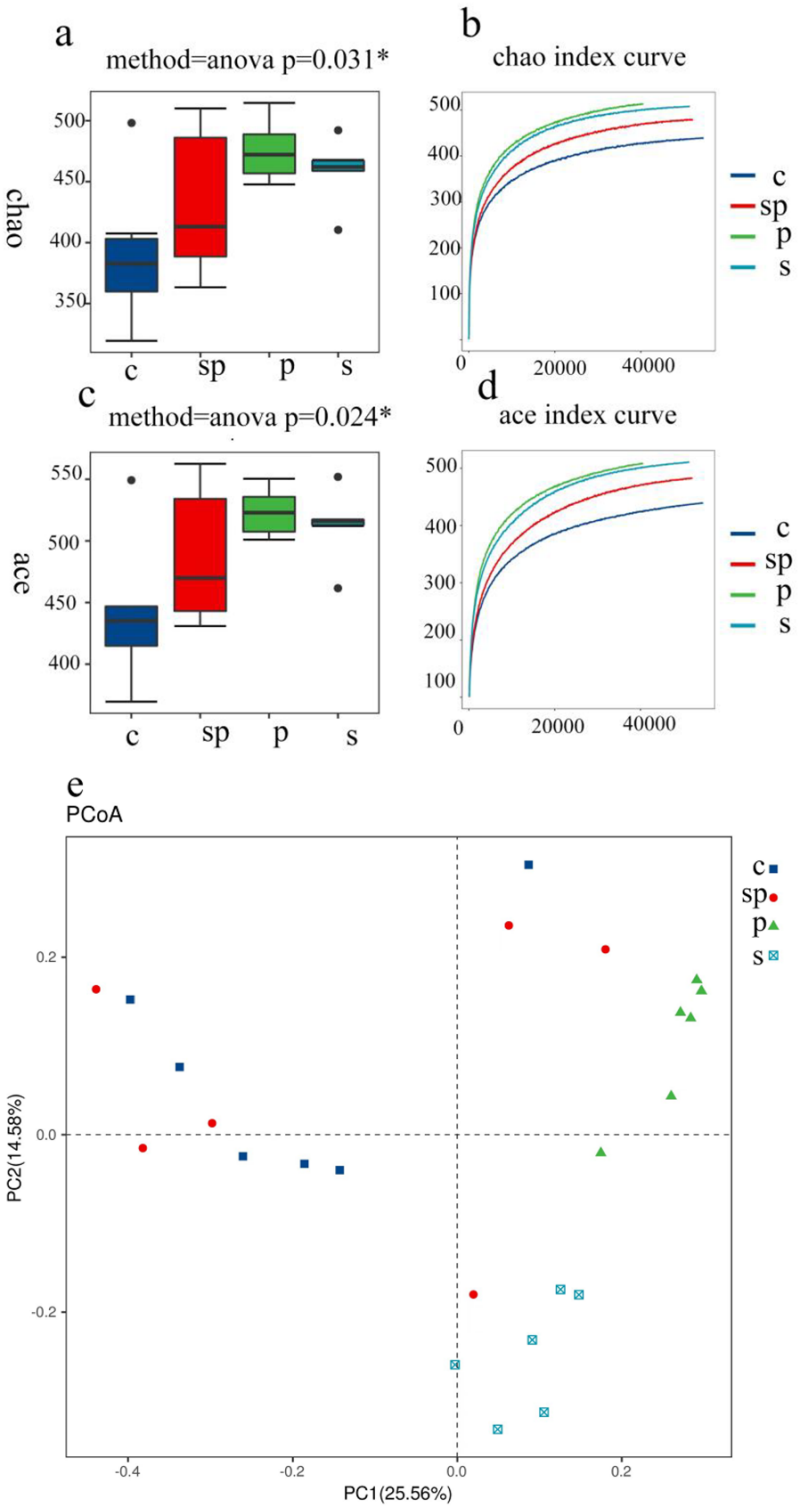
Differences in richness and diversity among groups. **a**, **b** Ace index results; **c**, **d** the Chao index results. **e** The variation analysis of PCoA among bacterial communities of all specimens, where each point represents an independent sample. C represents the control group; P represents the pyrotinib model group; S represents SBP group; SP represents the pyrotinib + SBP treatment group (The abbreviations for the following groups are the same)

In order to verify whether SBP's improvement of pyrotinib-induced diarrhea is related to intestinal microorganisms, we selected bacteria in cecal feces of the control group, the pyrotinib model group, the SBP group, and the pyrotinib + SBP treatment group for 16S rRNA V3-V4 region sequencing. Ace and Chao indices are commonly used to assess microbial α diversity [28]. Our results showed that Ace and Chao indexes were varied among different groups. The α diversity of the pyrotinib model group was significantly higher than that of the control group, while the α diversity of the pyrotinib + SBP treatment group was reduced (Fig. 4a–d). In addition, we used principal coordinate analysis (PCoA) of the bray-Bray-Curtis distance matrix [29] to further analyze the overall structural changes of intestinal flora between different groups and evaluate the variance of intergroup diversity. The PCoA diagram showed that the intestinal microflora of the pyrotinib model group and the control group were significantly separated (Fig. 4e). The bacterial members of the pyrotinib + SBP treatment group were highly similar to those of the control group. These results suggest that SBP for the treatment of pyrotinib-induced diarrhea might be related to the regulation of intestinal flora diversity.

#### SBP regulates the overall composition of the rat gut microbiome and key bacterial species

**Fig. 5:**
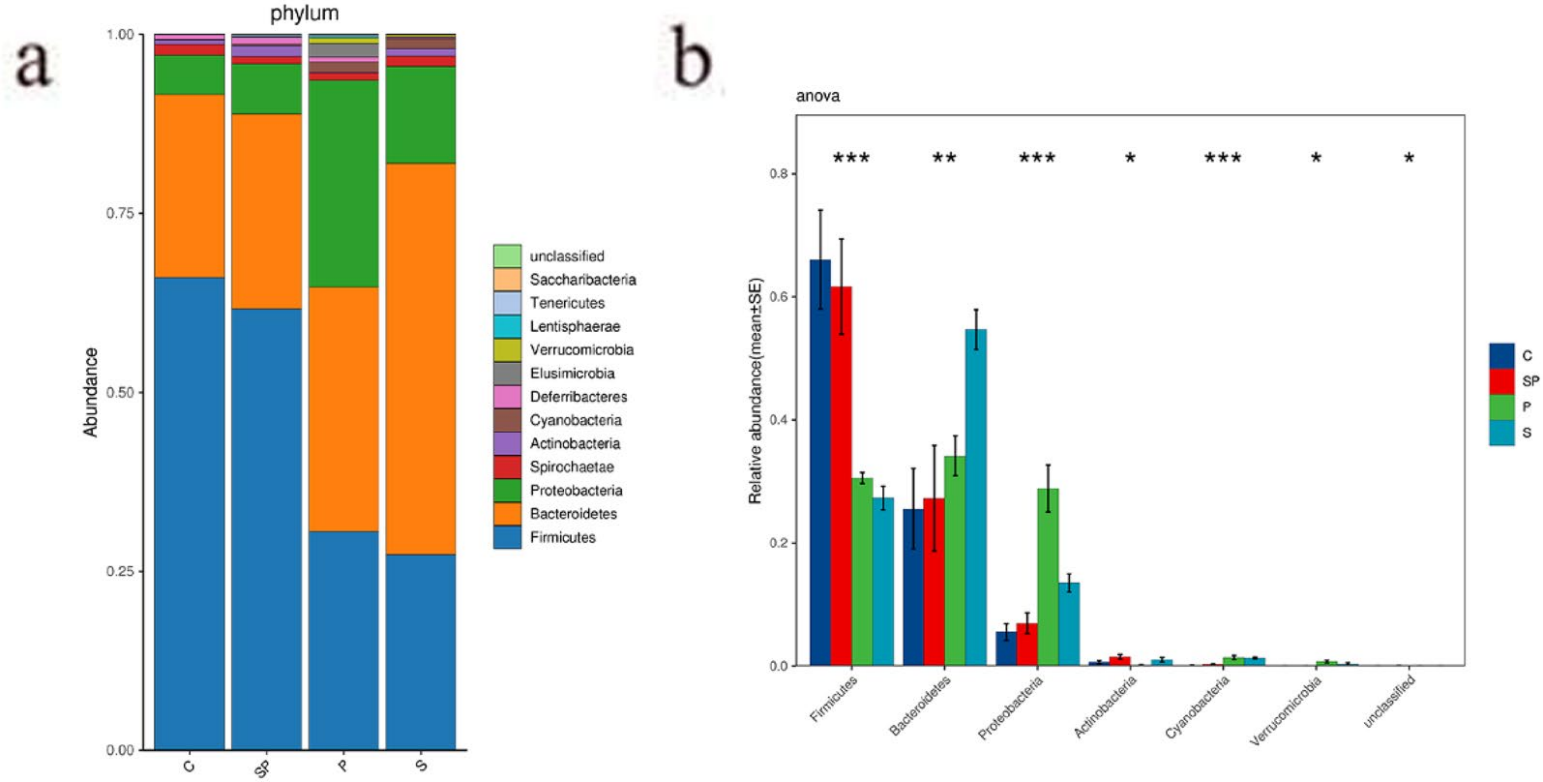
Results of 16S rRNA sequencing and Anova_Test. **a** The sequencing results of 16S rRNA. **b** The results of Anova_Test. C represents the control group; P represents the pyrotinib model group; S represents SBP group; SP represents the pyrotinib + SBP treatment group. * is a represent P < 0.05, and **represent P < 0.01; ***represent P < 0.001

The results of 16S rRNA sequencing showed that the most abundant phyla were detected in cecal fecal microflora, including Firmicutes, Bacteroidetes, and Proteobacteria. Anova Test was used to compare the differences in microbial composition among the four groups at different taxonomic levels. At the phyla level, the relative abundance of Proteobacteria in the pyrotinib model group significantly increased compared with the control group, while that of Firmicutes decreased significantly. The microflora structure of the pyrotinib + SBP treatment group was similar to that of the control group (Fig. 5a, b)*.* These experimental results suggest that the increased intestinal sensitivity caused by the large increase of harmful Proteus in the intestinal tract may be the main mechanism of diarrhea, while the inhibition of SBP on the growth of Proteus could be an important reason for improving diarrhea induced by pyrotinib.

**Fig. 6:**
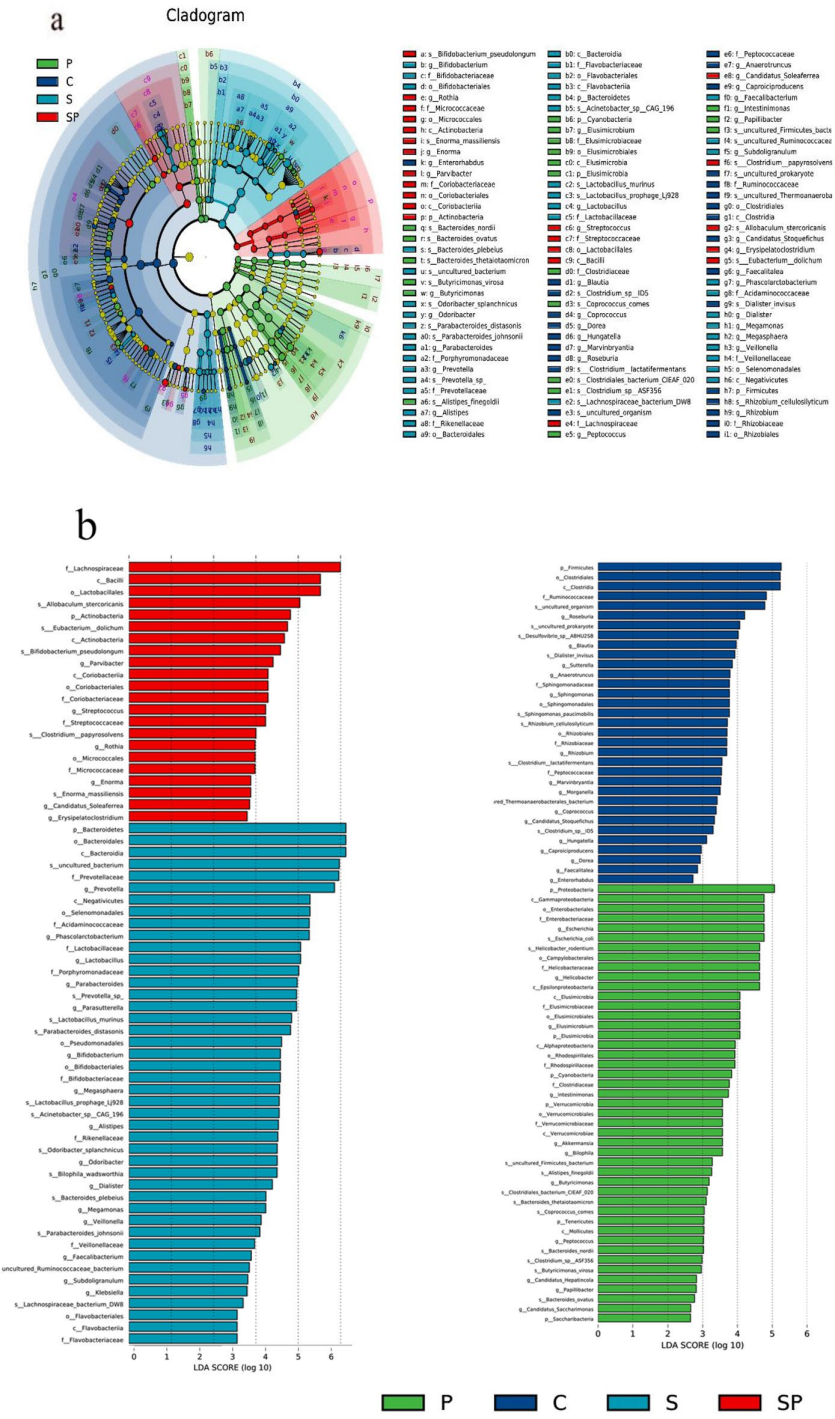
Taxonomic cladogram generated from LEfSe analysis of 16S rRNA gene sequences and the LDA scores of four groups were obtained by LEfSe analysis.** a** In a cladistic diagram, the circles radiating from the inside out represent the classification level from phylum to genus. The small circles in each classification represent subgroups of that order, and the diameter of the small circles is proportional to the relative abundance.** b** Histograms of the distribution of LDA values show organisms with large LDA scores, which are biomarkers for statistical differences between groups. The length of the histogram indicates the degree of influence of different organisms (LDA score)

To identify specific flora associated with SBP in the treatment of pyrotinib-induced diarrhea, linear discriminant analysis effect size (LEfSe) analysis was used to compare phylum to genus changes in the four groups [30]. There were significant differences in the composition of intestinal flora among all groups. The results showed that Firmicutes, Clostridiales, Ruminococcaceae and organism, LDA score (Log10)  > 4 were the most abundant in the control group. The proportion of opportunistic bacteria γ-Proteobacteria, Escherichia_coil, and Enterobacteriaceae, LDA score (Log10)  > 4 increased in the pyrotinib-induced diarrhea group. This significantly increased intestinal microbiota could be regarded as the biological manufacturer of pyrotinib-induced diarrhea. In the pyrotinib + SBP treatment group, the relative abundance of opportunistic bacteria decreased, and the relative abundance of Lachnospiraceae, Bacilli, Lactobacillales, and Allobaculum_ stercoricanis increased, LDA score (Log10)  > 4, which reshapes intestinal microbes to a certain extent (Fig. 6a, b).

### Analysis of ileum tissue metabolites in rats

**Fig. 7:**
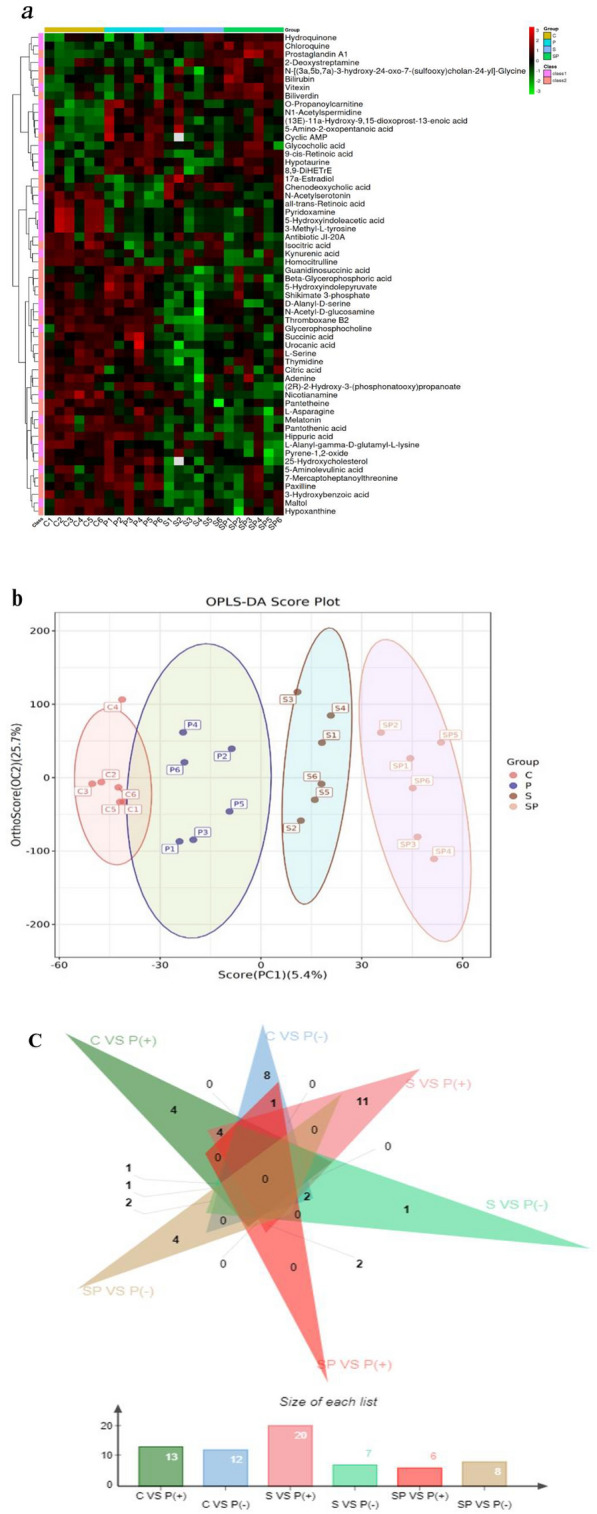
Hierarchical clustering heat map and OPLS-DA score map of differential metabolites. **a** Hierarchical clustering heat map of differential metabolites. The relative content in the figure is shown in different colors. The redder the color, the higher the expression level; the bluer the color, the lower the expression level. The columns represent samples, the rows represent metabolite names, and the cluster tree on the left of the figure is the differential metabolite cluster tree. **b** OPLS-DA score chart. The abscissa represents the first principal component interpretation degree, and the ordinate denotes the second principal component interpretation degree. Dots represent experimental samples, and colors symbolize different groups. The more concentrated the samples are within the group, the more scattered the samples are between the groups, indicating that the results are more reliable. **c** Comparison of differential metabolism of pyrotinib model group with the other three groups respectively. “ + ” represents the high content of differential metabolites in pyrotinib model group, and “−” represents the high content of differential metabolites in pyrotinib model group

As mentioned above, significant pathological changes occurred in the ileum of rats in the pyrotinib model group, and SBP could improve pathological damage to the ileum caused by pyrotinib. Therefore, ileum tissue samples of rats were taken for untargeted metabonomics analysis. A total of 532 metabolites were identified and quantified by ULTRA-performance liquid chromatography-tandem mass spectrometry (UPLC-MS/MS), among which 58 metabolites in all four groups showed significant differences (Fig. 7a). Moreover, the permutation test was conducted to verify that the intercept of Q2's regression line on the ordinate after OPLS-DA replacement test in ESI( +) and ESI(−) mode was all less than 0, indicating that there was no overfitting, and the OPLS-DA model was reliable. However, the intercept of Q2 regression line on the ordinate after OPLS-DA permutation test in ESI(-) mode was greater than 0, indicating that there was overfitting. The PLS-DA model is less reliable. Therefore, OPLS-DA model was used for the screening of metabolic biomarkers. (Fig. 7b).

**Fig. 8:**
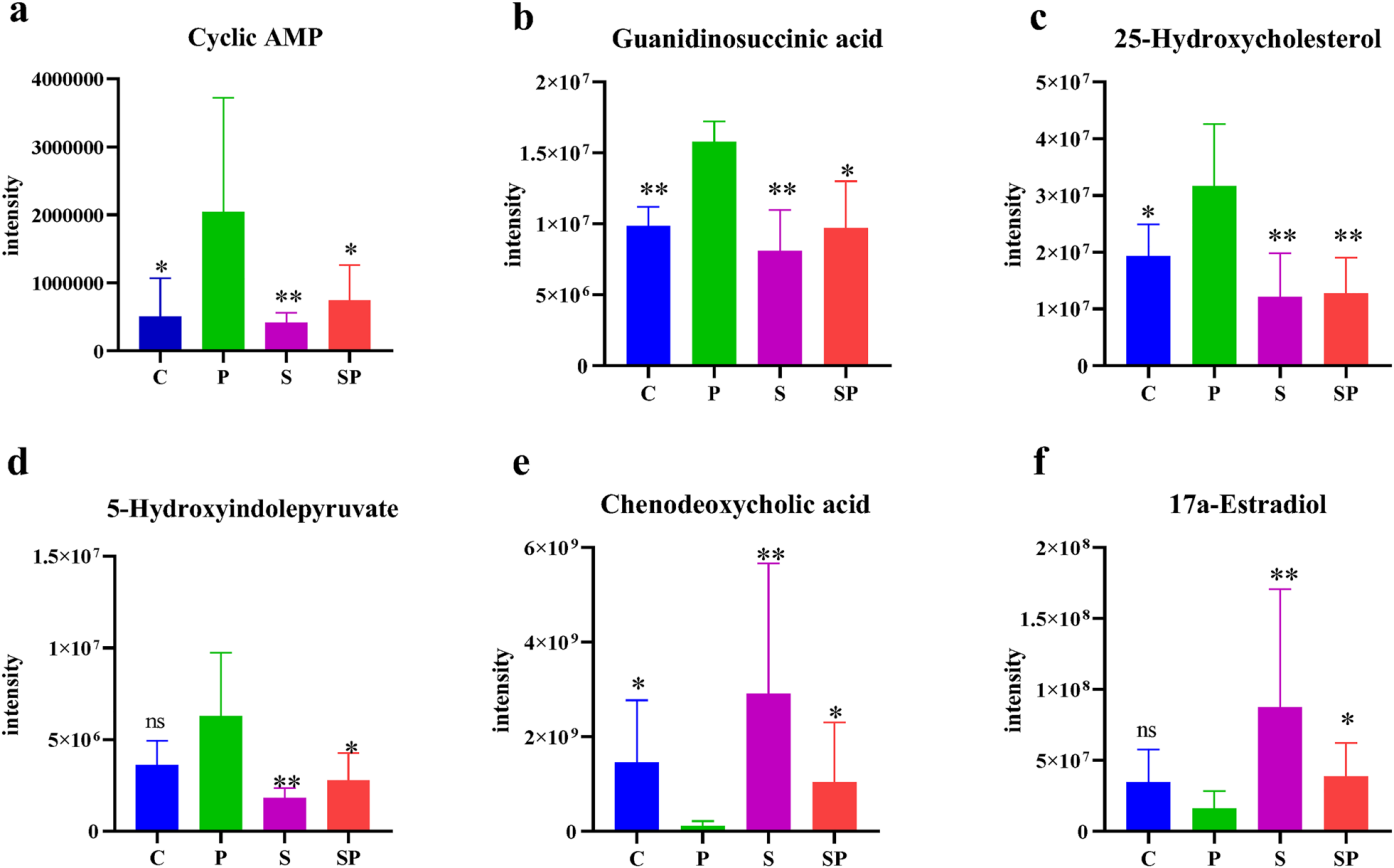
**a**–**g** Different metabolites in the four groups. The statistical difference among the groups was analyzed by One Way ANOVA. *P < 0.05; **P < 0.01; ***P < 0.001

**Fig. 9:**
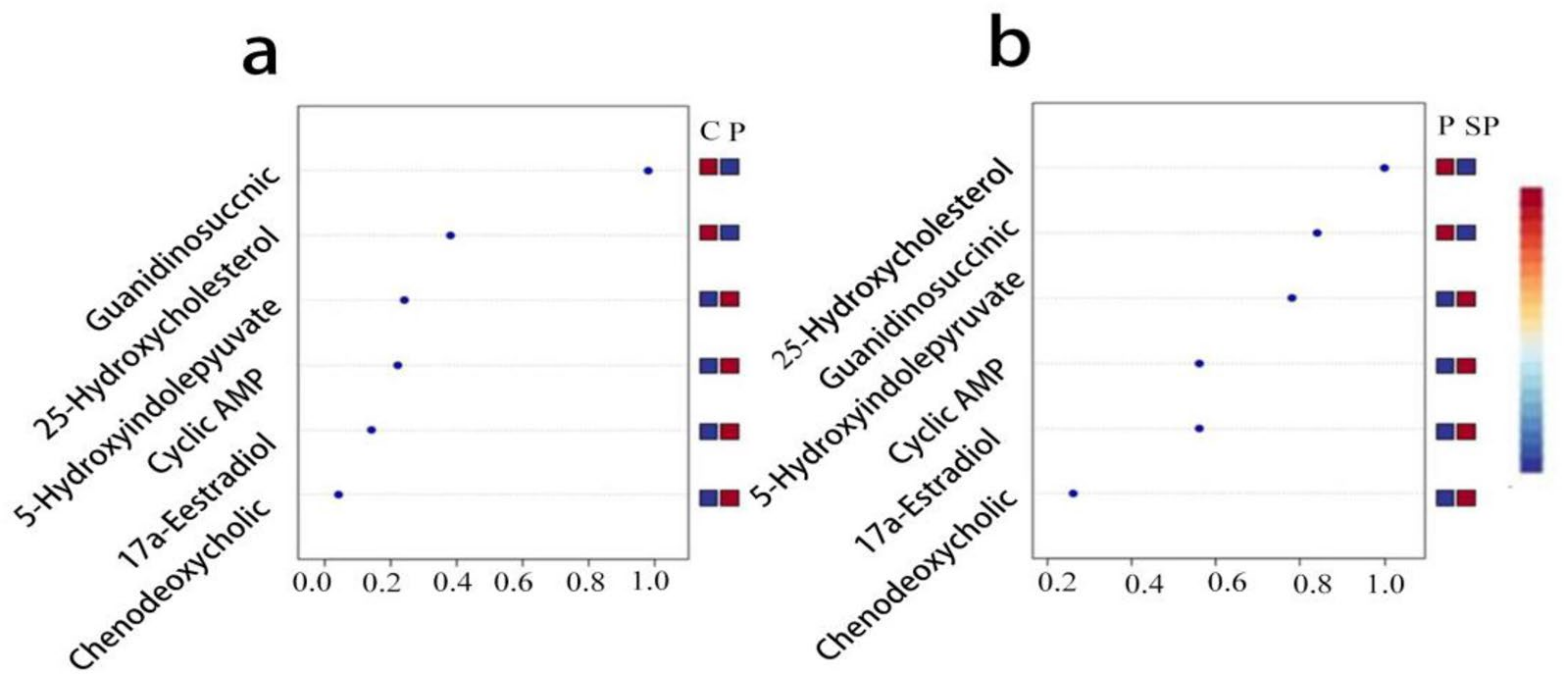
**a**, **b** Identification and trends of potential biomarkers in rats. Figure **a** shows that guanidinosuccinic acid, 25-hydroxycholesterol, 5-hydroxyindolepyruvate and cAMP were potential biomarkers in the control group and pyrotinib model group. Figure **b** shows that 25-hydroxycholesterol, guanidinosuccinic acid, 5-hydroxyindolepyruvate, and cAMP were potential biomarkers in pyrotinib model group and SBP treatment group. C: control group; P: pyrotinib model group; SP: pyrotinib + SBP treatment group

Based on VIP  > 1 and q-value  < 0.05, 25 metabolites were differentially expressed in intestinal tissues between the normal control group and the pyrotinib model group, among which 12 metabolites decreased and 13 metabolites increased in the pyrotinib model group. There were 14 differentially expressed metabolites in the pyrotinib model group and the pyrotinib + SBP treatment group, among which the content of 6 metabolites decreased and 8 metabolites increased in the pyrotinib + SBP treatment group (Fig. 7c). Six metabolites were identified as differential metabolites of SBP affecting pyrotinib-induced diarrhea (Fig. 8a–f), including cyclic AMP, chenodeoxycholic acid, guanidinosuccinic acid, 5-hydroxyindolepyruvate, 17a-estradiol, and 25-hydroxycholesterol. Consequently, an exploratory multivariate ROC curve analysis of metabolites was performed by MetaboAnalyst 5.0 system (https://www.metaboanalyst.ca/) [31]. The results suggested that 25-hydroxycholesterol, guanidinosuccinic acid, 5-hydroxyindolepyruvate, and cAMP might be employed as major potential biomarkers (Fig. 9a, b).

To explore the metabolic pathway of SBP in pyrotinib-induced diarrhea rats, MetaboAnalyst platform was used for functional enrichment analysis of DM (Differential metabolites). Based on pathway impact  > 0.03, the primary bile acid biosynthesis was identified as a potential metabolic pathway (Table 2).

**Table 2:** Metabolic pathway enrichment analysis

Pathway name	Match Status	p	-log(p)	Impact
Primary bile acid biosynthesis	2	0.012	1.90	0.011
Purine metabolism	1	0.235	0.63	0

The table below shows the detailed results from the pathway analysis. Since we were testing many pathways at the same time, the statistical p values from enrichment analysis were further adjusted for multiple testings

### Combined microbiome and metabonomics analysis

**Fig. 10:**
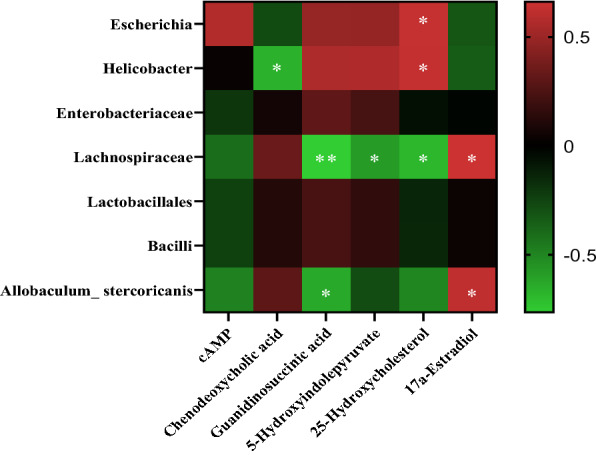
A heat map shows the correlation of intestinal tissue metabolites (ordinate) at the level of the intestinal microbiome family (ordinate). The degree of correlation is indicated by a gradient of red (positive) and blue (negative). *p < 0.05; **p < 0.01; ***p < 0.001; ****p < 0.0001

The influence of gut microbiota on host metabolic activity has been extensively studied, and it has an important impact on the host's local and systemic metabolism [32]. We established the correlation heat maps based on the Spearman correlation coefficient to explore the functional relationship between differential changes in gut microbiota and differential metabolites (Fig. 10). The decreased 25-hydroxycholesterol in the pyrotinib + SBP treatment group was negatively correlated with *Lachnospiraceae* and positively correlated with *Escherichia* and *Helicobacter*.

Guanidinosuccinic acid significantly decreased in the pyrotinib + SBP treatment group and was negatively correlated with *Lachnospiraceae and Allobaculum stercoricanis*. On the contrary, 17a-Estradiol significantly increased in the pyrotinib + SBP treatment group and was positively correlated with *Lachnospiraceae* and *Allobaculum stercoricanis*.

### Network pharmacology analysis

Network pharmacology was used to further explore the mechanism of SBP in treating pyrotinib-induced diarrhea. Firstly, the keyword “diarrhea” was searched from the Genecards database, and 1577 related genes (Relevance score  > 1) were retrieved. Secondly, by searching the TCMSP database, 285 effective compounds (OB ≥ 30%, DL ≥ 0.18) of SBP were selected, and 255 target genes of SBP were obtained by searching these effective compounds in the UniProt database. Finally, a Venn diagram was used for visualization processing, and 120 intersection targets were obtained (Additional file 1: Figure S1a*)*.

To identify the central gene of SBP in the treatment of diarrhea, we successfully constructed a PPI network map by importing the intersection target into the STRING database and importing Cytoscape 3.7.2 software for visualization processing. The PPI network diagram is shown in Additional file 1: Figure S1b. Seventeen targets were obtained by Network Analysis through intersection targets, including STAT3, RELA, JUN, TP53, AKT1, MAPK3, TNF, IL6, MAPK1, FOS, MAPK14, EGFR, STAT1, CTNNB1, IL10, MAPK8 and MYC (Degree > 18). The observed data suggest that these targets have a bridge role in treating pyrotinib-induced diarrhea with SBP.

In order to determine the anti-diarrhea function of candidate targets, we performed GO and KEGG pathway enrichment analyses by David. GO analyses included biological process (BP), molecular function (MF), and cellular component (CC). Pathways classified by BP category mainly included positive regulation of DNA-based transcription, positive regulation of RNA polymerase II promoter, positive regulation of PRI-miRNA transcription by RNA polymerase II promoter, positive regulation of gene expression, cell response to reactive oxygen species, and cell response to the cadmium ion (Additional file 1: Figure S2a). In addition, KEGG pathway analysis enriched 142 pathways (p < 0.05), where based on the correlation of the pathogenesis of diarrhea, the top 25 pathways were chosen as important pathways (Additional file 1: Figure S2b). Furthermore, combining our results with the mechanism of action of pyrotinib, inflammatory bowel disease, Toll-like receptor signaling pathway, IL-17 signaling pathway, ErbB signaling pathway, Pathogenic Escherichia coli infection, MAPK signaling pathway, cAMP signaling pathway and PI3k-Akt signaling pathway were chosen as potential pathways. These results suggested that combinations of herbs might have a wider range of possible molecular targets than each herb alone, which may help explain the synergistic effect of SBP action in treating pyrotinib-induced diarrhea [33].

## Discussion

Domestic and international clinical guidelines advocate symptomatic treatment with loperamide for TKI-induced diarrhea (Tyrosine Kinase Inhibitor). However, loperamide dosage is difficult to control and has been associated with adverse effects, such as constipation [34]. Furthermore, there is still no safe and effective treatment for TKI-diarrhea, especially pyrotinib-related diarrhea. This study determined SBP as a successful therapy for pyrotinib-related diarrhea.

Chinese medicine has shown promising results in the treatment of diarrhea. We found that SBP treatment reduced pathological intestinal damage caused by pyrotinib in rats, which is consistent with prior studies demonstrating that SBP exerts protective effects on the gastrointestinal mucosa [35]. Furthermore, BT-474 human breast cancer cell and Calu-3 human lung cancer cell with HER2 overexpression were selected for in vitro investigations. The results demonstrated that SBP could slightly increase the antitumor effect of pyrotinib. These findings imply that SBP is a safe and effective treatment for pyrotinib-induced diarrhea.

After verifying that SBP can alleviate pyrotinib-induced diarrhea without altering its anticancer activity, we collected gut microbiota from the cecum to investigate the mechanism of SBP. The intestinal microbiota has been recognized as a key pharmacological target of TCM. Studies have found that the distribution of intestinal microbiota can be altered by consuming TCM chemicals [14, 36]. The examination of gut microbiota revealed that SBP might dramatically lower the relative number of harmful bacteria Proteobacteria and regulate the relative abundance of Firmicutes to the same level as the control group (treated with saline). By modulating gut microbiota, these investigations revealed that SBP might have a role in reducing pyrotinib-induced diarrhea. In addition, LDA analysis revealed that the relative abundance of Lachnospiraceae and Lactobacillales was significantly higher in the pyrotinib + SBP treatment group compared with the pyrotinib model group, and there was a negative correlation between the relative abundance and the duration of host diarrhea, which was consistent with our previous findings [37]. Lachnospiraceae may affect the host epithelial cells and mucosal immune system in the mucosa, hence reducing the incidence of diarrhea [38]. Lactobacillales can regulate normal bacteria in the gastrointestinal tract, improve gastrointestinal function, prevent the growth of putrefactive bacteria in the intestinal system, and boost immunity [39]. Therefore, since SBP increases the relative abundance of Lactobacillales, it implies that SBP may be used to treat pyrotinib-induced diarrhea. Modern pharmacology shows that Poria cocos and its components can reduce the abundance of pathogenic bacteria such as Proteobacteria and Helicobacter, and promote the abundance of probiotics such as Prevotellaceae in mice with intestinal injury [40]. The application of atractylodes, the active component of atractylodes, could decrease the abundance of Helicobacter and Enterobacter, and increase the abundance of Lactobacillus [41]. Yam decreased the abundance of Blautia, Prevotella and increased the abundance of Lachnospiraceae, Clostridiales in rats with intestinal dysfunction [42]. In conclusion, some major herbs of SBP have regulatory effects on intestinal flora. Therefore, these evidences further indicate that SBP can treat pyrotinib-induced diarrhea by regulating intestinal flora disorder.

In this study, pathologically altered ileum tissue was taken to elucidate the relationship between metabolites and SBP in the treatment of diarrhea caused by pyrotinib. Based on OPLS-DA analysis and multivariate ROC curve analysis, we screened 4 major metabolites, i.e., 25-hydroxycholesterol, guanidinosuccinic acid, 5-hydroxyindolepyruvate, and cAMP. A previous study found that a high cholesterol diet inhibited the IgA response to the pathogen infection in mice, while CH25H deficiency enhances the IgA response to pathogen infection in mice [43], which is consistent with our results. Therefore, we speculated that SBP might enhance intestinal immunity through down-regulation of 25-hydroxycholesterol.

cAMP has been identified as a potential biomarker of adenylate cyclase activation in the epithelial membrane of the intestinal mucosa. Intracellular cAMP activation promotes the secretion of gastrointestinal water and electrolytes [44]. Our data suggested that cAMP levels were lower after SBP treatment, which may be due to the role of SBP in the treatment of pyrotinib-induced diarrhea by blocking cAMP activation. Subsequently, we made the metabolic pathways of the differential metabolites. The results showed that the differential metabolites were mainly enriched in primary bile acid biosynthesis. Bile acid metabolism is involved in the host- the interaction of microbes. Bile acid was synthesized from cholesterol in the liver and further metabolized through the gut microbiota [45]. At the same time, bile acid is an important signal molecule that is associated with FXR, FGFR4, and TGR5 bile acid receptors and can effectively regulate intestinal flora. It helps to maintain homeostasis of liver-intestinal circulation, as the change of intestinal flora directly affects the expression of FXR, FGF1-5, and other related genes [46, 47]. Therefore, it is crucial to explore the characteristics of bile acids in SBP treatment.

The gut microbiota has a crucial role in metabolic processes. In this study, we used multi-omics as a research tool to determine how SBP alters gut bacteria and tissue metabolism and elucidate the host effects of differential gut bacteria versus differential metabolites. Moreover, the correlation analysis between gut microbiota and metabolites revealed their relationship, thus further elucidating the pathogenesis of pyroitinib-induced diarrhea and the mechanism of SBP treatment [48, 49]. For example, given that *Lachnospiraceae*, a strain closely associated with diarrhea, which is found in the gut of most healthy people, could be a potentially beneficial bacterium [50]. We hypothesized that *Lachnospiraceae* might be involved in the mechanism of SBP in the treatment of pyrrotinib-induced diarrhea by affecting the metabolism of 25-hydroxycholesterol, guanidinosuccinic acid, and 5-hydroxyindolepyruvate.

To further understand the SBP in the treatment of diarrhea molecular model, a network between SBP active components, the corresponding targets, and diarrhea-related genes was established based on network pharmacology analysis [51]. The results of system network pharmacology analysis showed that SBP regulates multiple signaling pathways, all of which promote its therapeutic effect in the gut. In this study, we found that network pharmacology and intestinal microbes were connected with *Escherichia coli* infection, which further confirmed that SBP could improve the intestinal microenvironment by regulating pathogenic *Escherichia coli*, thus effectively treating diarrhea. Similarly, we found that toll-like receptor signaling could be focused on network pharmacology, metabolomics pathway and 25-hydroxycholesterol, as well as cAMP signaling pathway and cAMP, which confirms that SBP regulates 25-hydroxycholesterol and cAMP, thus exerting the anti-diarrhea effect.

## Conclusions

In summary, our study aimed to establish the internal relevance of metabolomics, intestinal flora, and network pharmacology. To the best of our knowledge, this is the first study to report the efficacy and safety of SBP in treating pyrotinib-induced diarrhea. We reported that SBP could effectively treat diarrhea caused by pyrotinib from the perspective of intestinal gut metabonomics and network pharmacology and explored the key targets and mechanisms of SBP in the treatment of diarrhea caused by pyrotinib. This study provides data and theoretical support for the in-depth study of this mechanism and lays a foundation for clinical application. However, further systematic molecular biology experiments are warranted to further explore the molecular process associated with SBP.

## Supplementary Information

**Additional file 1: Figure S1.** a-b Venn diagram of target genes related to pyrotinib-induced diarrhea treated with SBP(a). Intertarget interaction network of SBP in the treatment of pyrotinib-induced diarrhea(b). **Figure S2.** (a, b) GO enrichment analysis was performed on potential targets of main active ingredients from SBP against diarrhea. BP, biological processes; CC, cellular component.

## Data Availability

The data included in this investigation are available from the corresponding author. The authors confirm that the data supporting the findings of this study are available within the article and the raw sequencing results can be accessed with the accession number PRJNA858532. https://www.ncbi.nlm.nih.gov/sra/PRJNA858532.

## Abbreviations

SBP: Shenling Baizhu powder
TCM: Traditional Chinese medicine
EGFR: Epidermal growth factor receptor
HER2: Human epidermal growth factor receptor 2
AAALAC: Association for assessment and accreditation of laboratory animal care
IACUC: Institutional animal care and use committee
HE: Hematoxylin and eosin
LC–MS/MS: Liquid chromatography-tandem mass spectrometry
PCA: Principal component analysis
OPLS-DA: Orthogonal partial least square discriminant analysis
TCMSP: Traditional Chinese medicine
PPI: Protein–protein interaction
GO: Gene ontology
WB: Western blotting
PCoA: Principal coordinate analysis
LEfSe: Linear discriminant analysis effect size
DM: Differential metabolites
BP: Biological process
MF: Molecular function
CC: Cellular component
TKI: Tyrosine kinase inhibitor
