# Supplementary material for: Effects of Shenling Baizhu powder on pyrotinib-induced diarrhea: analysis of gut microbiota, metabonomics, and network pharmacology
Source: Chin Med. 2022 Dec 17;17:140. doi: 10.1186/s13020-022-00696-3 (PMC9759852; doi:10.1186/s13020-022-00696-3)
Supplement: Supplementary file 1 — Additional file 1: Figure S1. a-b Venn diagram of target genes related to pyrotinib-induced diarrhea treated with SBP(a). Intertarget interaction network of SBP in the treatment of pyrotinib-induced diarrhea(b). Figure S2. (a, b) GO enrichment analysis was performed on potential targets of main active ingredients from SBP against diarrhea. BP, biological processes; CC, cellular component. [file 13020_2022_696_MOESM1_ESM.docx]

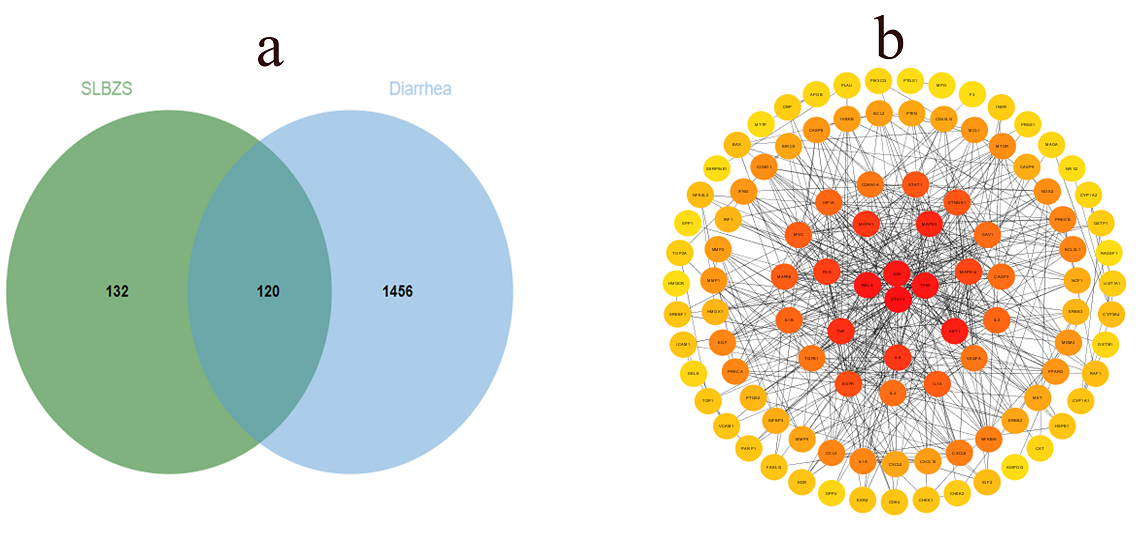


***Figure S1 a-b Venn diagram of target genes related to*** ***pyrotinib-induced diarrhea treated with SBP(a). Intertarget interaction network of SBP in the treatment of pyrotinib-induced diarrhea(b).***


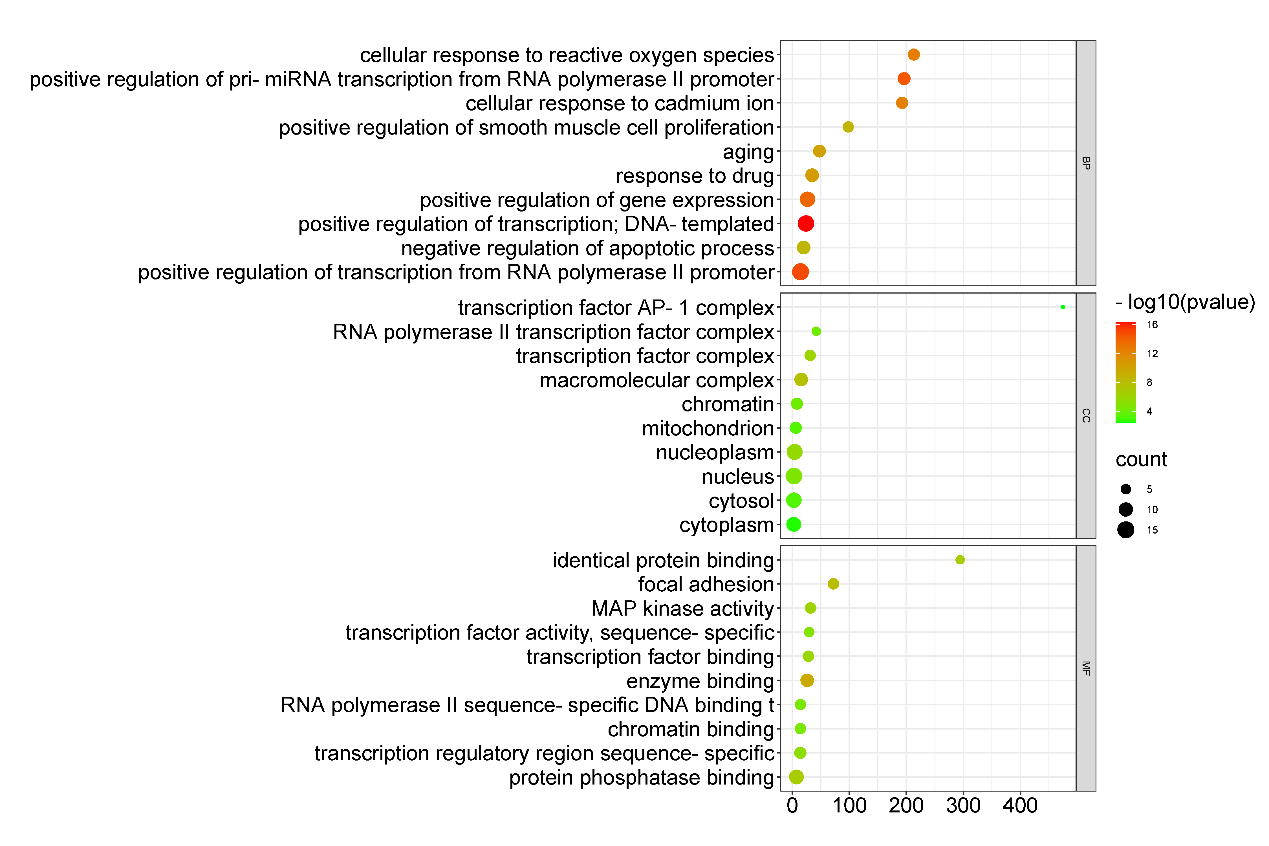


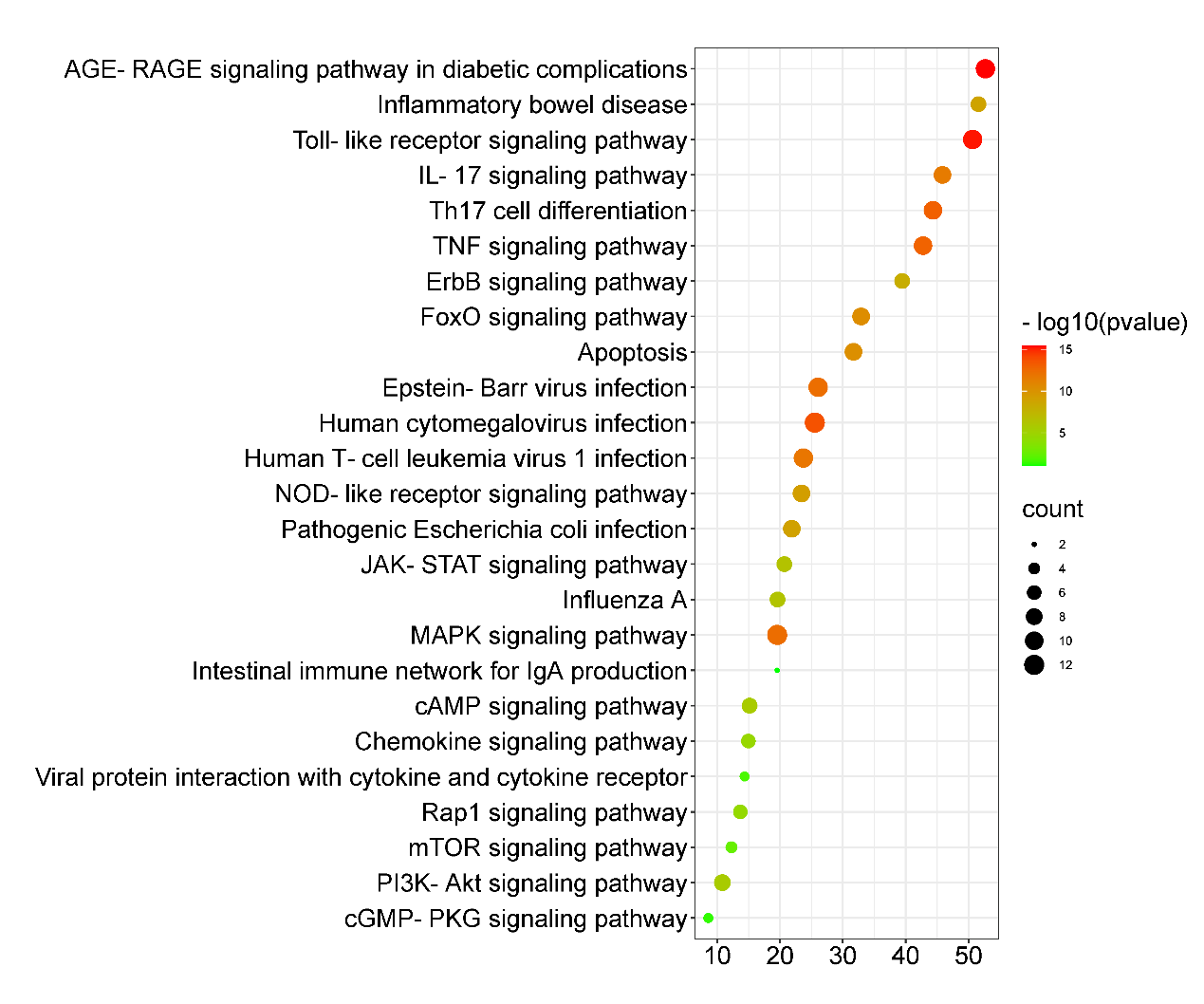


***Figure S2 (a, b)*** *GO enrichment analysis was performed on potential targets of main active ingredients from SBP against diarrhea. BP, biological processes; CC, cellular component.*
